# Integration of Bioinformatic Predictions and Experimental Data to Identify circRNA-miRNA Associations

**DOI:** 10.3390/genes10090642

**Published:** 2019-08-24

**Authors:** Martina Dori, Silvio Bicciato

**Affiliations:** Center for Genome Research, Department of Life Sciences, University of Modena and Reggio Emilia, Via G. Campi, 287, 41100 Modena, Italy

**Keywords:** circRNA, miRNA, target prediction, miRNA sponge

## Abstract

Circular RNAs (circRNAs) have recently emerged as a novel class of transcripts, characterized by covalently linked 3′–5′ ends that result in the so-called backsplice junction. During the last few years, thousands of circRNAs have been identified in different organisms. Yet, despite their role as disease biomarker started to emerge, depicting their function remains challenging. Different studies have shown that certain circRNAs act as miRNA sponges, but any attempt to generalize from the single case to the “circ-ome” has failed so far. In this review, we explore the potential to define miRNA “sponging” as a more general function of circRNAs and describe the different approaches to predict miRNA response elements (MREs) in known or novel circRNA sequences. Moreover, we discuss how experiments based on Ago2-IP and experimentally validated miRNA:target duplexes can be used to either prioritize or validate putative miRNA-circRNA associations.

## 1. Introduction

**Figure 1:**
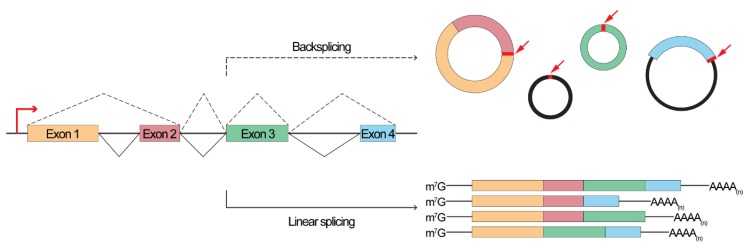
Linear vs Circular splicing. Circular RNAs (circRNAs) are formed from an unusual splicing event that results in covalently linked 3′–5′ ends termed as a backsplice junction (top, indicated by a red arrow). As for linear transcripts (bottom), circRNAs can undergo alternative splicing, resulting in different classes of transcripts (mono or multi exonic, intronic, exon-intron structure).

During the past decades, the field of RNA biology experienced an incredible evolution dictated by the discovery of long non-coding RNAs, the elucidation of the silencing pathways of short non-coding RNAs and, more importantly, of their regulatory functions [1]. Recently, a new class of non-coding RNAs has taken the scene: circular RNAs (circRNAs) [2]. Interestingly, the existence of RNAs with a circular form has been known for many years, but they were associated only with viruses and viroids (e.g., hepatitis δ virus [3]). Although few examples of circRNAs from transcribed genes were reported (e.g., Sry, [4,5,6], DCC [7], CYP450 [8]), only recently their abundance and regulatory functions have been described openly [9,10,11,12]. circRNAs consists in covalently closed RNA molecules with the 3′- and the 5′-ends linked in a non-collinear way resulting in the so-called backsplice junction (Figure 1) [13]. They result from an unusual splicing event that is believed to be mediated either by the pairing of long flanking introns (containing repetitive elements in an inverted orientation) or by an intra-lariat splicing [13,14,15,16,17,18,19]. As linear RNA, circRNAs can undergo alternative splicing that generates different classes of circRNAs (intron-containing, single exon, multiple exon, intergenic, intronic) and increases the “circ-ome” overall complexity [12,19,20,21,22,23]. This particular splicing event causes circRNAs to lack the 3′ poly(A) tail and the 5′ capping, a feature that confers resistance to exonuclease activity (e.g., RNase R [24,25]) and results, on average, in a longer half-life as compared to linear RNAs [11]. Since the first reports of circRNAs expression in humans and mice, thousands of potential circular RNAs have been predicted in different species (like Drosophila, *Caenorhabditis elegans* and plants) [9,11,12,15,26,27,28,29,30,31,32]. Despite the great attention that this elusive class of ncRNA has gathered, only a handful of transcripts have been fully functionally characterized. Nevertheless, the high stability combined with their identification in human body fluids (e.g., plasma [33] and saliva [34]) has greatly increased the interest toward circRNAs as potential disease biomarkers [35] and, following this idea, dozens of studies identified circRNAs in different pathological conditions [36] such as Alzheimer’s disease, atherosclerosis, myocardial infarction and, most importantly, cancer [37,38,39,40,41,42]. 

In this review, we explore one of the hypothesized functions for circRNA, i.e., miRNA binding. Particularly, we will first address the definition of “sponging”, which, so far, has been quite an appealing but misleading term, and then discuss the different computational approaches to predict miRNA-circRNA binding sites and the strategies to prioritize/validate such interactions.

## 2. To Sponge or Not to Sponge, That is the Question

**Figure 2:**
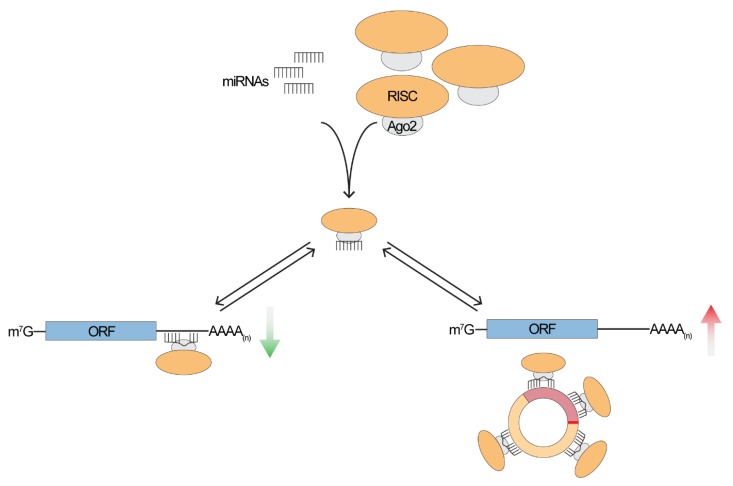
A miRNA-circRNA-mRNA network. It has been proposed that circRNA can act as a miRNA sponge, therefore competing with a linear target for the binding of the RISC complex. In the absence of circRNA, miRNAs are free to bind to their linear target, determining their repression. When the circRNA is expressed, the miRNA will guide the RISC complex to bind the circRNA, ultimately causing the de-repression of the mRNA. mRNA is depicted as an Open Reading Frame (ORF) with a 5′ cap (m^7^G) and a 3′ poly(A) tail.

**Figure 3:**
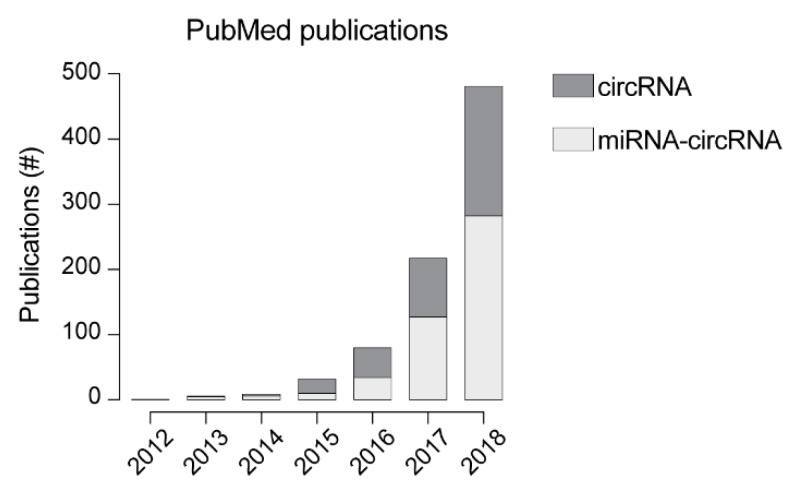
Per year number of publication indexed in PubMed. Dark grey represents the number of publications resulting by the search term “circRNA” while light grey represents the number of papers resulting from the combined search of “circRNA miRNA”.

The high stability and the presence in different body fluids make circRNAs extremely promising disease biomarkers. Given their diagnostic relevance, a lot of efforts have been put in the functional characterization of circRNAs, as this is critical to understand their role in disease development or progression and to provide crucial insights into their physiological role. It has been shown that nuclear circRNAs can be involved in regulating mRNA expression at the level of transcription by interacting with RNA polymerase or with members of the spliceosome machinery [43], for example. Conversely, cytoplasmic circRNAs seem to be involved in post-transcriptional regulation, sequester RNA-binding proteins [14,44] or even can be translated into small peptides [45,46]. Considering post-transcriptional regulation, one of the first and most investigated functions of circRNAs is miRNA sponging (Figure 2) [10,12,38,47,48]. In fact, in the past couple of years the number of papers involving circRNA-miRNA interaction has grown almost exponentially and, in 2018, represented ~60% of circRNA-related publications (Figure 3). Despite the increasing number of studies focusing on circRNA-miRNA interactions, to generalize this specific function to the entire “circ-ome” still remains challenging. The first, and most important, issue in this regard is the definition of “sponging” or, more appropriately, of competing endogenous RNAs (ceRNAs). Whether the ceRNAs hypothesis [49] is sufficient to explain the function of thousands of poorly characterized ncRNAs is still an argument worthy of great debate (refer to Thomson and Dinger [50] for more details). Nevertheless, it is crucial to consider the evidence that the expression alone of a ceRNA (in our case specifically, circRNAs) might not be sufficient to have a measurable effect on highly expressed miRNA and, therefore, on its downstream targets [51], while the impact on lowly expressed ones could be more significant [52]. This has a major consequence in the definition of the minimum characteristics that a circRNA must hold (e.g., expression, number of possible miRNA response elements—MREs—and miRNA expression itself) to be considered a miRNA sponge. Taking into account that, overall, circRNAs are expressed at lower levels than other RNAs [11,12,20,53] and that the expression is tissue- and cell-type-specific [20,23,30,54,55,56,57], the presence of a relatively high number of MREs for the same miRNA within the sequence of a single circular RNA would be expected. Different studies have shown that, beside CDR1as, only a very limited number of circRNA exhibit this property [30,53], pointing strongly toward the idea that sponging is an exception, rather than a general function.

## 3. Predicting circRNA-miRNA Binding Sites

Although circRNAs cannot be considered “sponges”, it is clear that these molecules fulfill their regulatory function also through the interaction with miRNAs [58,59,60,61,62,63,64,65]. To this end, knowing the sequence and the expression levels of circRNAs in a given tissue is essential. The most common approach to obtain this information is based on microarrays with probes specifically designed to target the most updated collection of human/mouse/rat circRNAs [66,67]. This allows the detection of even very lowly expressed circRNAs with high reproducibility (ideally, down to one copy), and facilitates the identification of differentially expressed circRNAs. Additionally, the development of more sophisticated RNA sequencing protocols (e.g., RPAD [68]) provides the possibility to identify highly pure circRNAs, overcoming the limit of relying only on annotated transcripts. Obviously, in the latter case, not only the sequencing protocol, but also which tool is used to identify the backsplice junction are critical issues and possible sources of variability between experiments [69]. No matter which approach is used for the identification of circRNAs, the bioinformatic prediction of MREs can be done in many different ways, mainly depending whether the circRNA is already known or a novel transcript.

### 3.1. Investigating Known circRNAs

In the past years, several databases have been released with the main goal of collecting all possible information regarding known circRNAs in different species (e.g., circBase [70], Table 1). These databases have expanded to meet the increasing complexity of circRNA expression patterns and to collect all possible information about functional predictions and associations with diseases (e.g., circNet [71], CircInteractome [72], circ2Traits [73]). One of the features that has been included is the miRNAs binding sites for all available circRNAs. These are obtained either using miRNA target prediction tools (as TargetScan [74], RNA22 [75], PITA [76], miRanda/miRSVR [77,78], etc.), like in the case of circNet and CircInteractome, or combining Ago binding sites with miRNA target predictions (as, for instance, starBase v2.0 [79,80] and its most updated version, ENCORI), although these approaches might result in multiple putative miRNAs hits per single circRNA. A possible strategy to reduce the number of candidate circRNA-miRNA associations is to consider also the downstream mRNAs (usually from databases including experimentally validated miRNA targets like TarBase v.8 [81] or miRTarBase [82]) and create a circRNA–miRNA–mRNA network. This procedure builds on the idea that an up-regulated circRNA will cause a down-regulation of its interacting miRNA that will ultimately determine an up-regulation of the target mRNA [83,84,85,86,87,88,89,90]. Networks that satisfy these expression criteria are selected finally for functional validation: first, the MREs predicted within the circRNA are validated primarily by luciferase assay and then the expression of the target miRNA and mRNA are evaluated upon circRNA depletion (or overexpression, according to the initial transcription pattern). Although quite successful, this approach presents some limitations, for instance, the databases providing miRNA binding predictions are dealing only with human circRNAs, with the exception of starBase [79,80] which includes also data for mouse and *C. elegans*. Additionally, this workflow is effective only when the circRNA has been previously identified and annotated in other databases (like circBase) and, finally, it also requires a differential expression analysis for all components of the network (circRNA, miRNA and mRNA). Moreover, validating MREs with luciferase assay has two major drawbacks: i) it does not always provide a clear proof of direct interaction [91]; and ii) it implies that the MREs on the circRNA have to be sufficiently strong to cause a significant variation in either luminescence or luciferase mRNA levels, which is not necessarily the case. These issues can be overcome by an RNA Immunoprecipitation assay (RIP, [92]) that will provide information of what is directly binding to the circRNA, with no regard to a functional output [93,94,95,96,97,98,99,100].

### 3.2. Characterizing Novel circRNAs

As mentioned previously, circRNAs have been shown to have a time- and tissue-specific expression pattern [20,23,30]. This results in the need, due to the complexity of organisms, to perform comprehensive assessments to investigate specific tissues and developmental stages. The best way to address this issue is through RNA sequencing, since this method is not limited by an a priori knowledge of circRNA sequences and expression. In turn, when it comes to MREs prediction, the bioinformatic approach becomes less straightforward. Assuming that the full sequence of novel circRNAs has been assessed, the first issue is represented by the choice of an appropriate tool to predict MREs. Although it has been shown that more than 80% of circRNAs are overlapping coding genes, less than 10% include a 3′UTR, making it almost useless to take advantage of available databases (like TargetScan [74], Table 2) that contain information on 3′UTRs only. On the other hand, databases that provide information on MREs on the entire sequence (e.g., microRNA.org [101]) and also include experimental validation information (like TarBase v8 [81] or STarMirDB [102]) do not allow to browse by target sequence in addition to gene name, therefore becoming useless for the analysis of novel circRNAs. There are some tools that have been designed to also search by custom sequences (e.g., STarMir [103]), but they show limitation in the length of the queried sequence, allowing the analysis only of few transcripts and making it difficult to perform a comprehensive assessment. One way to overcome these limitations is to use the stand-alone versions of the algorithms behind the prediction database (when available). Thus, the direct application of the algorithms allows the analysis of any given sequence for any given list of miRNAs. Unfortunately, results obtained with this approach show an extremely high rate of false positives, requiring either a systematic validation of the targets (e.g., with RIP assays) or the integration with known interactions.

## 4. Integrating Seed Prediction on Custom Sequences with Experimental Data

**Figure 4:**
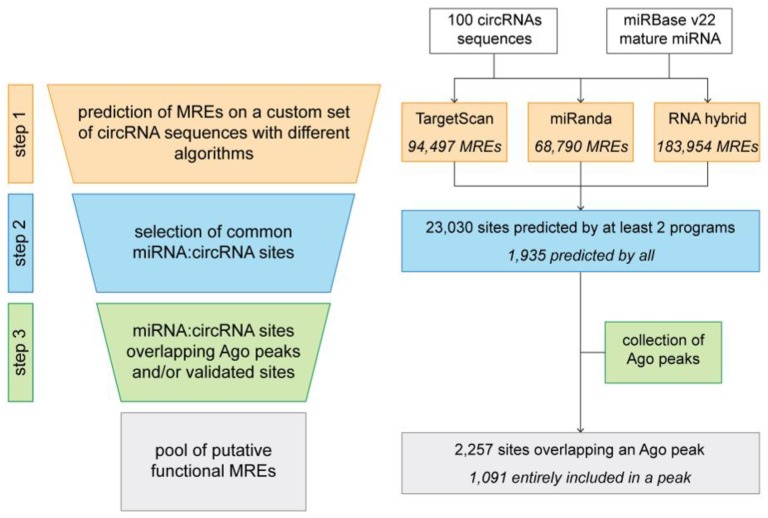
A possible pipeline for the comprehensive assessment of circRNA:miRNA binding sites starting from a custom set of expressed circRNA sequences (left) and a practical example on the outcome of each step on a set of randomly chosen sequences from previous work [30] (right).

No matter which method is used to design the prediction algorithm, the major reason behind the high rate of observed false positives and false negatives is the fact that miRNA-target recognition already is effective with a seed length of six nucleotides [104,105]. Reducing and prioritizing the predicted interaction is not trivial, as each possible approach has several pros and cons. For example, to consider conservation of seed and MREs across species dramatically reduces the number of predictions, although this approach does not consider non-canonical, as well as non-conserved, binding sites [106]. Moreover, using the free energy of miRNA:target duplexes is effective at the cost of an incredibly high number of putative very stable false positive interactions. Given that no gold standard has been identified nor do any algorithms outperform the others, there are some steps that can be undertaken to “manually” predict circRNA-miRNA sites while limiting the possibly overwhelming list of predicted binding interactions. This workflow (depicted in Figure 4, left) can be divided into three main steps. To show how each step influences the final outcome, we analyzed 100 randomly chosen mouse circRNA from previous work [30]. As done in some databases for gene-miRNA target mining (e.g., miRWalk [107]), the first step consists in performing the analysis with the same input (circRNAs) and miRNA sequences using different algorithms (for this example, TargetScan [74], miRanda [77] and RNAhybrid [108] were used, Figure 4, right). Using default options, we obtained for our cohort of circRNAs an average of 115,747 putative MREs where RNAhybrid predicted the highest number of sites (183,954) while miRanda the lowest (68,790). The second step consists in retrieving only the predictions that have been identified by at least half of the programs (in our case we selected MREs predicted by at least two programs). This first filtering step reduced the initial list to approximately 23,000 MREs, with only 1935 sites predicted by all programs. In particularly, for TargetScan only ~19% of predicted sites were common to at least another algorithm, while for miRanda the sites were reduced to approximately 30%. Regarding RNAhybrid, we observed the most severe reduction, as only 5% of all the predicted MREs were kept for the last step. To further reduce the amount of possible false positives, a valid approach is to make use of complementary experimental data. Specifically, since the binding of the RISC complex is mediated by the interaction of the miRNA with members of the Argonaute protein family [109,110], it is fundamental, for a predicted miRNA-circRNA site to be real, that Ago proteins also are binding in the same positions. The development of various CLIP-Seq protocols (Cross-linking and Immunoprecipitation followed by sequencing) provides an extremely valuable source of high-throughput data of Ago binding sites [111,112,113,114,115,116,117]. These data can be directly used to eliminate all the predicted sites for which there is no binding of Ago protein [118], considering this a sine qua non condition for a true binding of miRNA on the target circRNA. Considering this, the last step consists in the retrieval of all the MREs that are overlapped also by Ago and to this end we used a collection of publicly available Ago-binding sites from mouse brains [115,119]. We obtained a final set of 2257 sites partially overlapping an Ago peak and among these, 1091 MREs were included entirely in a peak. Using this final filtering, we could reduce the number of putative circRNA:miRNA sites down to 0.9% and 1.5% for TargetScan and miRanda predictions, respectively. Again, the most dramatic decrease was observed for RNAhybrid predictions as only 0.2% of MREs were included in the final list. Since Ago CLIP-Seq experiments are not available for all cell types, tissues and organisms, for this last step accessible data also can be used indirectly, for instance, by creating a pool of Ago-binding motifs and exploiting sequence similarity to quantify existence probability of custom miRNA:circRNA duplexes.

### Ranking MREs

The approach presented in this example uses a basic step-wise filtering system that takes the output of different prediction programs and sequentially reduces the pool of MREs according to the presence/absence of specific criteria (prediction by at least 2 out of 3 algorithms; overlap with an Ago peak). As shown, this system already is effective in reducing the amount of data that can be considered for further validation and functional characterization, but we still might be missing valuable information given by the pool of MREs that are specific to each algorithm but that still overlap an Ago peak (~12,000 additional MREs in total). To overcome this limitation, a possible alternative is the construction of a scoring function that evaluates the probability of a predicted MRE to be real by considering the validated data (presence of an Ago peak/binding motif) and the initial prediction information (observation that the same site is identified by one or more algorithms) [120]. Weighting these two aspects differently, the resulting score would allow the ranking of all predicted MREs and to prioritize those that include both the experimental data and the predictions by multiple algorithms while not excluding all the sites identified by single programs that still retain a correspondence among validated data. 

## 5. Concluding Remarks

Circular RNAs have recently emerged as a novel class of transcripts characterized by covalently linked 3′–5′ ends called backsplice junctions. Studies have shown their relevance in physiological and pathological conditions, in particular as disease biomarkers and potential therapeutic targets. However, the functional characterization of these sequences is still in its infancy and the role of relatively few circRNAs has been described to date. Recently, great effort has been put toward understanding one specific mechanism, i.e., miRNA sponging. Due to the combined overall low expression of circRNAs and the low number of MREs predicted within their sequence, only a handful of transcripts really can be considered true “sponges”. Nevertheless, it is undeniable that circRNAs are capable of binding miRNAs but, more than a “sponge” for a single small RNA, they might function as a scaffold for several different ones. Regarding this, it is crucial to develop appropriate pipelines that allow a more accurate prediction of the miRNA targets, thus facilitating an overall assessment of miRNA binding and, possibly, leading to the identification of a more general function.

## Figures and Tables

**Table 1:** List of relevant circRNA-related databases including the available organisms and their general features.

Database	Website	Organisms	Features
**circBase**	http://www.circbase.org	Human Mouse Fly Worm Fish Planaria	Most updated catalogue of predicted circRNAs. Beside human and mouse, it also collects data from several other organisms
**circInteractome**	https://circinteractome.nia.nih.gov	Human	Enables the prediction and mapping of binding sites for RNA binding proteins and miRNA on known circRNAs. It includes also a module for siRNA design for knock-down experiments and primer design for PCR
**circNet**	http://syslab5.nchu.edu.tw/CircNet/	Human	Provides tissue-specific expression patterns, integrated miRNA-circRNA-mRNA networks, circRNA isoform expression and genomic annotation
**ENCORI** **StarBase v2**	http://starbase.sysu.edu.cn/index.php http://starbase.sysu.edu.cn/starbase2/index.php	Human Mouse Worm	Designed for investigating interaction networks of lncRNAs, miRNAs, ceRNA, RNA binding proteins and mRNAs from public CLIP-Seq data. It also allows to browse for circRNA-miRNA interactions.
**circ2Traits**	http://gyanxet-beta.com/circdb/	Human	Link of circRNA with disease inferred by miRNA-disease associations

**Table 2:** List of the most common databases and algorithms for predicting miRNA binding sites together with the organisms for which the prediction can be browsed (by sequence and/or by gene ID) and if they share the standalone version.

Tool	Website	Organisms	Browse by Sequenc/Gene ID	Standalone Version
**STarMir**	http://sfold.wadsworth.org/cgi-bin/starmirtest2.pl	Human Mouse Worm Other	Sequence/ Gene ID	no
**STarMirDB**	http://sfold.wadsworth.org/starmirDB.php	Human Mouse Worm	Gene ID	no
**PITA**	https://genie.weizmann.ac.il/pubs/mir07/index.html	Human Mouse Fly Worm	Gene ID	yes
**miRanda/** **mirSVR**	http://www.microrna.org/microrna/home.do	Human Mouse Rat Fly Worm	Gene ID	yes
**TargetScan**	http://www.targetscan.org/vert_72/	Human Mouse Fly Worm Zebrafish	Gene ID	yes
**RNAhybrid**	https://bibiserv.cebitec.uni-bielefeld.de/rnahybrid/	Any	Sequence	yes
**TarBase v8**	http://carolina.imis.athena-innovation.gr/diana_tools/web/index.php	Human Mouse Rat Chicken Zebrafish Fly Worm Chimpanzees Macaque Soy Maize Barrelclover Grape wine Earthmoss Epstein–Barr virus KSHV	Gene ID	no
